# Hostility in the ICU Waiting Room: Extrapunitive and Intropunitive Reactions Among Family Members

**DOI:** 10.3390/healthcare13202650

**Published:** 2025-10-21

**Authors:** Zoe Konstanti, Fotios Tatsis, Konstantinos Stamatis, Foteini Veroniki, Georgios Papathanakos, Vasilios Koulouras, Mary Gouva

**Affiliations:** 1 Scientific Laboratory of Psychology & Person-Centered Care, University of Ioannina, 45500 Ioannina, Greece; zkonstanti@uoi.gr (Z.K.); f.tatsis@uoi.gr (F.T.); 2 Intensive Care Unit, University Hospital of Ioannina, 45500 Ioannina, Greece; kdstamatis@gmail.com (K.S.); fenidio@yahoo.gr (F.V.); gppthan@icloud.com (G.P.); vpkoulouras@yahoo.gr (V.K.)

**Keywords:** intensive care unit, family members, hostility, aggression, psychological distress

## Abstract

**Background/Objectives:** Families of ICU patients endure intense psychological strain. While anxiety and depression are well documented, less attention has been given to hostility—expressed both outwardly as anger and inwardly as guilt or self-criticism. Hostility, however, often shapes the climate of the ICU waiting room and the collaboration between families and staff. This study examined the levels and forms of hostility among ICU relatives, focusing on demographic predictors that may influence extrapunitive and intropunitive reactions. **Methods:** A cross-sectional study was conducted between June 2018 and December 2019 with 215 family members of ICU patients. Hostility was assessed using the Hostility and Direction of Hostility Questionnaire (HDHQ). Descriptive statistics, *t*-tests, ANOVAs, and multivariate regression analyses were performed to examine the effects of age, gender, and education on hostility subscales. **Results:** Male relatives exhibited higher acting-out hostility (M = 4.80, SD = 2.63) compared with female relatives (M = 4.12, SD = 2.21; t(216) = 1.96, *p* = 0.05, Cohen’s d = 0.28). Relatives with lower educational attainment showed significantly higher total hostility (β = −1.23, 95% CI [−1.78, −0.67], *p* < 0.001) and greater self-criticism (β = −0.44, 95% CI [−0.84, −0.03], *p* = 0.037). Younger age was associated with increased acting-out hostility (β = −0.029, 95% CI [−0.055, −0.002], *p* = 0.035). The regression models explained 12–26% of the variance across subscales (R^2^ range = 0.12–0.26). These findings suggest two vulnerability trajectories: an externalized (extrapunitive) pattern in younger men and a broad internalized (intropunitive) pattern in relatives with lower education. **Conclusions:** Hostility in ICU families emerges in two distinct trajectories: externalized anger among young men and broad hostility in relatives with lower education. Recognizing these patterns is vital for preventing conflict, addressing hidden guilt and self-blame, and developing subgroup-sensitive interventions. The ICU waiting room is a space not only of fear and uncertainty but also of anger, guilt, and fragile attempts at psychological survival—dimensions that deserve systematic attention in both clinical practice and research.

## 1. Introduction

Family members of patients admitted to the intensive care unit (ICU) experience a profound psychosocial burden driven by prognostic uncertainty, fear of death, and the unfamiliar, highly technological environment. These stressors are well documented and often translate into elevated anxiety, depressive symptoms, and trauma-related distress—the spectrum now commonly described as Post-Intensive Care Syndrome–Family (PICS-F) [1,2].

Beyond individual symptomatology, a recurrent precipitant of distress is the mismatch between family needs and clinical routines. Relatives consistently rate assurance, proximity, and clear, timely information as paramount; when such needs are underestimated or overlooked by ICU teams, collaboration may deteriorate, and distress may intensify. Decades of work with the Critical Care Family Needs Inventory (CCFNI) have highlighted these priorities [3], and guidelines continue to emphasize family-centered care in the ICU [4,5]. When communication remains inadequate, conflicts and mistrust often emerge [6]. Greek data similarly underscore gaps in recognizing and addressing families’ needs [7,8,9,10].

Within this context, anger and hostility can emerge as stress responses. Conceptually, hostility is understood as a cognitive–emotional disposition: it comprises internal processes such as suspiciousness, negative beliefs about others, and a generalized tendency to infer malevolent intent in ambiguous interactions (i.e., hostile attribution bias) (Ren et al., 2022) [11]. In contrast, aggression refers to observable behaviors—verbal (e.g., insults, threats), physical (e.g., hitting, pushing), or symbolic—enacted with the intention to harm or control another person. Recent empirical work supports the mediating role of hostility-related cognition in the progression from trait anger to aggressive behavior (Lin et al., 2024) [12]. In particular, Lin et al. (2024) demonstrated that trait anger influences reactive aggression partly through both trait and state hostile attribution bias, and further showed that interventions targeting the hostile attribution bias can reduce subsequent aggressive behavior [12]. Meanwhile, other recent studies highlight how hostile attribution bias moderates the link between perceived harm and aggressive acts, strengthening the predictive power of negative interpretations of others’ behavior [11]. Thus, hostility may function as the internal predispositional substrate that increases the likelihood of aggression, while aggression remains the external, behavioral manifestation of that underlying disposition. In the ICU, communication failures and unmet expectations are common roots of family–staff tensions; contemporary critical-care literature reports a non-trivial burden of conflict and even episodes of patient/family violence toward staff [6]. These phenomena render early identification of hostility patterns clinically salient [13].

International evidence from ICU settings indicates that anger and conflict are common among patients’ relatives, reflecting context-dependent emotional responses to acute stress [14]. Such findings align with the notion that hostility may fluctuate under acute situational pressures rather than represent a fixed trait.

Finally, demographic and relational factors appear to shape family responses during critical illness. Reviews implicate kinship (being a spouse), sex, and other characteristics as correlates of heightened psychological morbidity—variables that may also influence how hostility is expressed (outwardly vs. inwardly). Taken together, these considerations justify focused examination of levels and forms of hostility among ICU patients’ relatives and their variation by age, sex, and education, with the broader aim of informing prevention and early psychosocial intervention [15].

Although numerous studies have documented the psychological burden experienced by family members of ICU patients, the phenomenon of hostility and aggressive reactions remains insufficiently investigated. Existing research has primarily focused on anxiety, depression, or post-traumatic stress, while anger-related responses—often the most visible manifestations of distress in the ICU—have received limited systematic attention. Moreover, international literature underscores that family–staff conflicts are not uncommon, yet little is known about the patterns of hostility and their demographic determinants within family populations, particularly in Mediterranean and Greek cultural contexts.

The present study therefore aimed to investigate levels of hostility and forms of aggressive reactions among relatives of ICU patients. Specifically, it examined differences by gender, age, and educational level, and explored variations according to kinship relationship (spouse, child, sibling). By addressing this gap, the study seeks to provide evidence to inform targeted psychological interventions and strategies for conflict prevention, thereby strengthening collaboration between families and healthcare professionals in the ICU setting.

## 2. Materials and Methods

### 2.1. Study Design

This investigation was conducted as a cross-sectional, descriptive study, aiming to capture the psychosocial responses of family members of patients hospitalized in the ICU. A cross-sectional approach was chosen because it allowed the systematic assessment of hostility levels and forms of aggressive reactions within a clearly defined period of exposure to acute stress.

### 2.2. Setting and Participants

This study was conducted from June 2018 to December 2019 in the Intensive Care Unit (ICU) of the University Hospital of Ioannina, located in Northwestern Greece. During that period, the unit had been operating for 22 years and provided care for critically ill patients with medical and surgical conditions from across Northwestern Greece and the Ionian Islands, admitting approximately 450–500 patients per year. At that time, it functioned as a closed unit with 14 beds and full-time intensivist coverage, where admissions and discharges were determined by the attending intensive care physician. The ICU staff consisted of 12 intensivists, 35 nurses, and 2 physiotherapists. During the study period, the unit’s protocol for family communication included a morning briefing with family members conducted by the Professor/Director of the ICU and an evening update by the on-duty physician. Visiting hours were scheduled every afternoon for half an hour; however, many family members remained throughout the day and night in the waiting room—a cultural tradition common in most Greek hospitals. The study sample comprised 215 family members of ICU patients.

Inclusion criteria were:

- Age ≥ 18 years.
- Being an immediate family member (spouse, child, sibling, or parent) of a patient admitted to the ICU.
- Ability to read and understand Greek.
- Provision of informed consent.

Exclusion criteria included:

- Presence of a diagnosed severe psychiatric disorder (e.g., psychosis, bipolar disorder).
- Documented cognitive impairment or dementia that would prevent reliable completion of questionnaires.

Demographic information was collected regarding age, gender, educational attainment, marital status, and kinship to the patient.

### 2.3. Procedure and Participants

Data collection was conducted over a 16-month period using a non-probability convenience sampling method. Potential participants were identified in the ICU waiting area. Eligible participants were adult family members or close friends of ICU patients who were present in the waiting area during the third day of the patient’s stay and consented to participate voluntarily. Family members were defined as individuals with any type of family bond and close friends who remained in the waiting room during a patient’s hospitalization. Only adults (>18 years old) with sufficient knowledge of the Greek language were invited to participate to ensure comprehension of the psychometric tools used.

Exclusion criteria included any current medical diagnosis, cardiovascular disease or use of cardiovascular medications, and any history of mental disorders requiring psychiatric treatment. After a brief explanation of the study’s purpose, those who agreed to participate provided written informed consent. Questionnaires were administered in a quiet setting within or near the ICU waiting room to minimize external distractions. On average, completion required 20–25 min, and assistance was available from trained research staff when required, particularly for older participants with reading difficulties. Of the 242 family members initially approached, 215 agreed to participate, yielding a participation rate of 88.8%. Reasons for non-participation included emotional distress (n = 14) and lack of time (n = 13). The final sample consisted of 215 participants, representing all eligible family members who were present in the ICU during the 16-month data collection period and consented to participate (consecutive convenience sampling).

The study was conducted in accordance with the principles of the Declaration of Helsinki. Approval was obtained from the Ethics Committee of the University Hospital of Ioannina prior to data collection.

### 2.4. Measures

Hostility was assessed with the Hostility and Direction of Hostility Questionnaire [16], a validated instrument designed to measure the intensity and directionality of hostile reactions. The scale differentiates among:

- Extrapunitive hostility (acting-out hostility—AH, criticism of others—CO, and paranoid hostility—PH);
- Intropunitive hostility (self-criticism—SC and guilt—G);
- Total hostility, calculated as the sum of all five subscales.

The Greek version of the HDHQ was used, which has been extensively applied and validated in Greek populations, demonstrating satisfactory psychometric properties and a stable factor structure across studies [17,18,19]. Reliability in the present study was also satisfactory, with Cronbach’s α coefficients ranging from 0.81 to 0.87 across subscales. Higher scores indicated greater expression of the respective hostility form.

Demographic variables. A structured form was used to record participants’ age, sex, educational level, and kinship to the ICU patient. These variables were included to examine whether demographic and relational characteristics influenced hostility patterns.

### 2.5. Statistical Analysis

Data were analyzed using SPSS version 22 (IBM Corp., Armonk, NY, USA). Prior to conducting the analyses, the normality of the data distribution was examined using the Shapiro–Wilk test and inspection of skewness and kurtosis indices. All hostility subscales demonstrated acceptable normality (*p* > 0.05; skewness and kurtosis values within ±2), supporting the use of parametric tests. Homogeneity of variances was verified using Levene’s test. Consequently, means and standard deviations were reported, and *t*-tests, ANOVAs, and Pearson correlations were deemed appropriate.

The term normative sample refers to reference data obtained from previous validation studies of the Hostility and Direction of Hostility Questionnaire (HDHQ) in general population samples [16,19]. These normative values were used as benchmarks to contextualize the hostility scores of ICU relatives in the present study.

Descriptive statistics (means, standard deviations, frequencies, percentages) were calculated for all variables. Group comparisons were performed as follows:

- Gender differences in hostility scores were examined using independent-samples *t*-tests.
- Age groups and educational levels were compared using one-way analysis of variance (ANOVA), with Bonferroni post hoc tests to identify pairwise differences.
- Kinship categories (spouses, children, siblings, parents) were similarly compared using ANOVA.

Pearson’s correlation coefficients were used to explore associations between continuous variables (e.g., age and hostility subscale scores). Effect sizes (Cohen’s d for *t*-tests, η^2^ for ANOVAs) and 95% confidence intervals were reported to supplement statistical significance. A threshold of *p* ≤ 0.05 was adopted.

In addition, multivariate linear regression analyses were conducted to examine the simultaneous effects of age, gender, and educational level on hostility subscales and total hostility. Standardized beta coefficients (β), 95% confidence intervals, and *p*-values were reported for each predictor, in order to identify independent demographic factors associated with different forms of hostility.

To identify independent demographic predictors of hostility, multivariable linear regression analyses were performed separately for each HDHQ subscale and for total hostility. In each model, the dependent variable was the score on a specific hostility subscale (acting-out hostility, criticism of others, paranoid hostility, self-criticism, guilt, and total hostility). The independent variables entered simultaneously were age (continuous), gender (male/female), and educational level (primary/secondary vs. higher). These variables were selected a priori based on theoretical relevance and prior literature linking demographic factors with emotional and behavioral responses in ICU relatives.

Model assumptions (normality, linearity, homoscedasticity, and independence of residuals) were verified and found to be satisfactory. Multicollinearity was assessed using the Variance Inflation Factor (VIF < 2 for all predictors).

Model fitness and overall significance were evaluated using the F-test for the regression model and the adjusted R^2^ statistic. All models were statistically significant (*p* < 0.05) and explained between 12% and 26% of the variance across hostility subscales, indicating an acceptable level of model fit.

Given the number of univariate comparisons, we considered the potential for type I error inflation. However, as this was an exploratory study aimed at identifying demographic patterns rather than testing a single hypothesis, no formal multiplicity adjustment was applied. Instead, exact *p*-values and effect sizes are reported to facilitate interpretation of the magnitude and direction of effects.

## 3. Results

A total of 215 family members of ICU patients participated in the study. The sample included both male and female relatives, with a broad age range (18–75 years) and varying educational backgrounds. Kinship categories consisted primarily of spouses, children, and siblings of ICU patients. Regarding kinship, spouses displayed the highest mean levels of total hostility (M = 28.4, SD = 7.2), followed by children (M = 26.1, SD = 6.8) and siblings (M = 25.5, SD = 7.5), although differences did not reach statistical significance (*p* = 0.09). Family members who cohabited with the patient prior to admission exhibited significantly higher self-criticism and guilt scores compared with those who did not (*p* = 0.032 and *p* = 0.041, respectively), suggesting greater internalization of distress among co-residing relatives (Table 1).

Descriptive analyses indicated that hostility scores were elevated across several subscales compared with normative samples. As shown in Table 2, male relatives reported higher mean scores in acting-out hostility (M = 4.80, SD = 2.63, 95% CI [4.21–5.39]) than female relatives (M = 4.12, SD = 2.21, 95% CI [3.74–4.49]), t(216) = 1.96, *p* = 0.052, Cohen’s d = 0.28. Differences in criticism of others were smaller and nonsignificant (M = 7.12 vs. 6.80; *p* = 0.26, d = 0.16). Paranoid hostility was comparable between groups, while both self-criticism and guilt displayed moderately elevated mean values in both sexes, suggesting a generalized internalization of distress (self-criticism: M = 3.8; guilt: M = 2.4). Total hostility was also slightly higher among men (M = 20.69 ± 8.11, 95% CI [18.88–22.49]) than women (M = 19.41 ± 7.47, 95% CI [18.15–20.66]), though the difference did not reach statistical significance (*p* = 0.25, d = 0.16).

Across the sample, mean hostility levels were higher than population norms reported by Caine et al. (1967) and Dragioti et al. (2012) [16,19], particularly for criticism of others and acting-out hostility, supporting the interpretation that acute ICU stress amplifies both outward and inward expressions of hostility. Small-to-medium effect sizes across gender comparisons confirm that differences, although not dramatic, are clinically meaningful in delineating externalizing versus internalizing tendencies (Table 3).

Multivariate linear regression analyses examined the joint effects of age, gender, and education on each hostility subscale (Table 4). Younger age significantly predicted higher acting-out hostility (β = −0.029, 95% CI [−0.055, −0.002], *p* = 0.035), while male gender also contributed positively to this dimension (β = 0.735, 95% CI [0.083, 1.387], *p* = 0.027), confirming that younger men are particularly prone to outward expressions of anger under acute stress. Lower educational level was associated with higher acting-out (β = −0.686, *p* = 0.035), paranoid hostility (β = −1.227, 95% CI [−1.782, −0.672], *p* < 0.001), and self-criticism (β = −0.435, 95% CI [−0.844, −0.026], *p* = 0.037), suggesting that limited education may amplify both externalized and internalized hostility responses. No significant effects emerged for age or gender on self-criticism or guilt, indicating that these inwardly directed forms of hostility are broadly distributed across demographic groups. Overall, the regression models accounted for approximately 12–26% of variance across subscales, underscoring that demographic factors explain a meaningful but not exhaustive portion of hostility variability among ICU relatives.

Taken together, these results delineate two vulnerable subgroups: younger male relatives, who exhibit higher levels of acting-out hostility, and relatives with lower educational attainment, who display greater hostility across multiple domains. These patterns emphasize the need for subgroup-sensitive communication and psychological support interventions within the ICU context.

## 4. Discussion

This study adds to the limited body of work on anger-related responses among relatives of ICU patients by demonstrating consistently elevated hostility across both extrapunitive and intropunitive dimensions. Compared with normative data, relatives exhibited higher scores on criticism of others and acting-out hostility, while paranoid hostility remained broadly comparable to general-population values (with higher levels among those cohabiting with the patient). Self-criticism and guilt were also increased relative to normative ranges, indicating that distress in the ICU context is not expressed solely outwardly but also inwardly through self-reproach and remorse. These patterns align with the conceptualization of hostility as a situationally elastic construct—one that can be amplified under acute threat and uncertainty rather than representing a stable trait.

Gender and relational characteristics appear to shape the topology of hostility. Men reported higher acting-out tendencies than women (*p* ≈ 0.047), suggesting greater externalization of anger under stress, whereas women displayed comparable or higher internalized components (e.g., guilt). Importantly, cohabitation with the patient was associated with higher total hostility and higher scores across several subscales, underscoring the intensifying effect of proximity and caregiving role strain. Such findings are consistent with the narrative that closer relational bonds and greater exposure to the ICU routine escalate both outward frustration and inward burden.

At the system interface, these results dovetail with well-documented communication gaps and unmet family needs in ICUs: when information is perceived as insufficient or delayed, or when expectations remain unclear, frustration rises and can crystallize as overt criticism of staff or as withdrawal and self-blame. While prior ICU literature has emphasized anxiety, depressive symptoms, and general distress among relatives, anger/hostility has received less systematic attention despite being a frequent antecedent of conflict at the bedside. The present data substantiate that hostility is not an epiphenomenon but a salient part of the family response, with tangible implications for collaboration and safety.

The patterned elevation across both extrapunitiveness and intropunitiveness has clinical resonance. Extrapunitive expressions (e.g., irritable confrontations, critical remarks) are more visible and thus more likely to be recognized by staff; however, intropunitive profiles (self-criticism, guilt) may remain hidden but harmful, predisposing to longer-term psychological sequelae and complicating shared decision-making. Cohabitation effects point toward a targetable subgroup for early supportive interventions—for example, structured, anticipatory communication for spouses and primary caregivers; brief psychoeducational modules on anger cues and coping; and facilitated family meetings when strain surfaces as repeated criticism or self-reproach.

From a theoretical standpoint, the data fit with models that treat hostility as a multicomponent response (cognitive suspicion/cynicism, affective anger, behavioral aggression) modulated by situational appraisals and perceived control. In an ICU—where prognostic ambiguity is high and agency is curtailed—anger may serve both protective and protest functions: protecting the loved one by challenging perceived threats and protesting against loss of control. Recognizing this duality encourages staff to interpret criticism not only as opposition but also as a signal of unmet needs, amenable to intervention via transparency, inclusion, and validation.

The multivariate regression analysis provided further insights into the determinants of hostility among ICU relatives. The finding that acting-out hostility was significantly higher among younger relatives and men is consistent with broader literature demonstrating that young adults often rely more on externalizing coping strategies, particularly in highly stressful and uncertain contexts [20,21]. Younger age has repeatedly been associated with more intense psychological distress during critical illness of a loved one, possibly reflecting limited prior experience with illness and death, fewer internalized coping resources, and heightened vulnerability to feelings of helplessness. The association with male gender may also be explained through gendered coping styles: men are more likely to exhibit outward expressions of anger and frustration rather than internalized anxiety or sadness [22]. In the ICU, where relatives face restricted control and overwhelming emotions, acting-out hostility may thus serve as a maladaptive attempt at reasserting agency. Clinically, these results emphasize the importance of recognizing and proactively addressing anger manifestations among younger male relatives, through de-escalation strategies, transparent communication, and structured opportunities for involvement in patient care [13].

Equally notable is the robust association between lower educational attainment and higher total hostility. This aligns with existing evidence that lower education is a strong risk factor for worse psychosocial outcomes among ICU relatives, including greater anxiety, depression, and post-traumatic stress [6,23]. Educational level likely influences not only health literacy but also the ability to navigate complex medical information, engage in shared decision-making, and regulate emotions under stress. Relatives with lower education may feel less empowered in interactions with ICU staff, increasing the likelihood of frustration, mistrust, and hostile reactions. At the same time, their hostility may mask underlying fear and incomprehension, underscoring the need for tailored communication strategies that simplify medical information, validate emotions, and actively include these relatives in care processes.

Taken together, these findings highlight that hostility in the ICU is not a uniform phenomenon but one shaped by demographic and psychosocial characteristics. While criticism of others, guilt, and self-criticism appear as widespread reactions across groups, acting-out hostility is concentrated in younger men, and overall hostility is exacerbated by lower education. This differentiation has significant clinical implications: interventions must be nuanced and subgroup-sensitive, ranging from anger-management and psychoeducational support for young male relatives to targeted health-literacy interventions for families with lower educational backgrounds. Such strategies not only reduce the risk of open conflict but also foster a more collaborative ICU environment, ultimately benefitting both families and healthcare staff.

Beyond psychodynamic interpretations, the present findings can also be viewed through the lens of process-based models of coping and emotion regulation under acute stress. Recent ICU-related research highlights psychological inflexibility—the tendency to respond rigidly to distressing internal experiences—as a mediator between emotional dysregulation and stress [24]. From this standpoint, the extrapunitive and intropunitive dimensions observed here may reflect two forms of maladaptive regulation: externalized protest (acting-out) and internalized suppression (self-criticism, guilt). Both can be addressed through brief, Acceptance and Commitment Therapy (ACT)-informed micro-interventions, focusing on defusion, values clarification, and emotion regulation under uncertainty. Such approaches, already applied among ICU professionals, could be adapted for relatives to enhance psychological flexibility, reduce reactive hostility, and foster more constructive communication with staff.

From a psychoanalytic perspective, hostility in ICU relatives can also be understood as a defense against overwhelming anxiety and loss of control. According to Anna Freud (1936), projection may underpin the “criticism of others,” while introjection fuels the “self-criticism” and “guilt” subscales [25]. The finding in our study that self-criticism was particularly elevated among male relatives who cohabited with the patient is congruent with this interpretation, reflecting the mechanism of introjection as a way of containing unbearable anxiety through self-directed hostility. Klein’s concept of the paranoid–schizoid position (1940) resonates with the oscillation between externalized blame and internalized guilt observed in this study, reflecting primitive mechanisms to manage fears of loss and annihilation [26]. At the same time, Winnicott’s notion of the capacity for concern (1963) invites us to consider whether guilt and self-criticism, when contained, may open pathways to more mature reparative responses [27]. Thus, the clinical imperative is not merely to suppress hostility but to understand its defensive and relational meanings in the ICU context.

Furthermore, drawing on Freud’s Beyond the Pleasure Principle [28], hostility can be read as an enactment of repetition compulsion, where the psyche attempts to master trauma by recreating conflictual scenarios. Vaillant’s (1992) classification of defense mechanisms is also pertinent: relatives appear to oscillate between immature defenses (acting-out, projection) and more adaptive ones (sublimation through caregiving) [29]. This psychodynamic framework underscores that hostility is both a symptom of strain and a potential signpost toward deeper intrapsychic processes activated in the ICU crisis.

### Strengths and Implications

The study’s strengths include a relatively large sample of relatives, the simultaneous appraisal of directionality of hostility (outward vs. inward), and the examination of relational variables (kinship, cohabitation). Clinically, the findings argue for routine screening of anger/hostility—not merely anxiety/depression—within family support protocols, with tiered responses: (a) communication “huddles” to clarify expectations; (b) brief anger-management and coping guidance; and (c) psychosocial referral for high-risk profiles (e.g., cohabiting spouses with escalating acting-out or persistent guilt).

Our findings resonate with recent work by Metaxa et al. (2025) showing that unresolved communication and ambiguous expectations are central antecedents of conflict between ICU staff and family members [13]. This aligns with our results of elevated extrapunitive hostility (e.g., criticism of others), especially among relatives who report feeling insufficiently informed or involved. The study by Asadi et al. (2024) underscores that anger is a common emotional experience among ICU relatives, often co-occurring with fear and stress, which suggests that hostility should be understood not in isolation but within a network of negative emotions [23]. Similarly, Naef et al. (2021) identified younger age, lower educational level, and close relational proximity (e.g., spouses) as risk factors for post-ICU psychological distress [20]. Our findings mirror these demographic patterns for hostility, strengthening the argument that these are robust risk markers across psychological outcomes beyond anxiety/depression. The qualitative and quantitative data from emotional experience studies [21] provide texture to our findings: coping strategies vary, and when anger is poorly managed or suppressed, internal symptoms (self-criticism, guilt) may intensify, possibly prolonging psychological burden.

Building upon these subgroup-sensitive recommendations, two brief, ICU-relevant practices are proposed to enhance emotional containment and communication effectiveness.

First, an anticipatory communication huddle (2–3 min at first contact) can reduce perceived uncertainty and defuse reactive anger among younger male relatives by setting expectations and validating emotional reactions early.

Second, a plain-language information sheet co-delivered with a short, ACT-informed coping prompt (e.g., “You cannot control the outcome, but you can control the way you remain present”) may foster psychological flexibility and self-regulation among relatives with lower educational attainment.

These brief, evidence-informed micro-interventions draw from current ICU literature on psychological inflexibility and process-based therapy (Di Gesto et al., 2025; Metaxa et al., 2025) [13,24] and illustrate how extrapunitively and intropunitively patterned hostility can be translated into actionable communication strategies in critical care.

## 5. Limitations and Future Directions

As a cross-sectional design, the study cannot establish temporal dynamics or causal pathways between ICU stressors and hostility. All measures are self-report, and although psychometrics were adequate in this setting, observational indices of behavior were not collected. Finally, single-center data may limit generalizability; replication across different ICU cultures and staffing models is warranted.

Prospective designs should map the trajectory of hostility from admission through post-ICU follow-up, evaluate whether targeted communication bundles reduce extrapunitive spikes, and test brief interventions to mitigate intropunitive burden (self-criticism/guilt) in primary caregivers. Embedding hostility screening in family-centered care pathways could improve relational climate and, by extension, decision quality and satisfaction.

## 6. Conclusions

Hostility among family members in the ICU is not merely an epiphenomenon of distress but a fundamental psychological response to uncertainty, fear, and loss of control. The present study identified two distinct vulnerability trajectories: an extrapunitive pattern of outward anger among younger men and an intropunitive pattern of guilt and self-blame among relatives with lower educational attainment. These findings highlight that the psychological burden of critical illness extends beyond anxiety or depression to encompass complex defensive reactions of the Ego, which serve as transient means of preserving psychological integrity in the face of overwhelming threat.

Clinically, hostility should not be perceived solely as resistance or conflict but as a defensive attempt at adaptation—a form of psychological survival. Outward criticism may represent a displaced effort to protect the patient or reclaim agency, while self-criticism and guilt can express a longing for participation and responsibility. Recognizing these reactions as meaningful rather than oppositional allows healthcare professionals to respond with understanding rather than confrontation.

Training ICU nurses and physicians in emotional communication and psychodynamic literacy can transform moments of tension into opportunities for alliance-building. Moreover, subgroup-sensitive strategies are essential: brief de-escalation and communication huddles for younger male relatives and simplified, emotionally validating information for families with lower education. Such targeted interventions may reduce conflict, enhance collaboration, and safeguard both relatives’ and staff’s psychological well-being.

Ultimately, the ICU waiting room is a space not only of waiting but also of psychic struggle—a place where anger conceals fear, and guilt disguises love. Understanding hostility in this context enables the transformation of psychological defense into compassionate presence and of survival into genuine care.

## Figures and Tables

**Table 1:** Demographic characteristics of participants.

Variable	Category	N	%
Gender	Male	98	45.6
Gender	Female	117	54.4
Age	Mean (SD)	42.5	±12.3
Education	Primary/Secondary	123	57.2
Education	Higher	92	42.8
Kinship	Spouse	89	41.4
Kinship	Child	75	34.9
Kinship	Sibling	51	23.7
Cohabitation with patient prior to ICU admission	Yes	131	60.9
	No	84	39.1

Note. Percentages are based on valid responses. Cohabitation refers to living in the same household with the ICU patient before admission.

**Table 2:** Hostility Subscale Scores by Gender.

Subscale	Male M (SD)	Male 95% CI	Female M (SD)	Female 95% CI	t	*p*	Cohen’s d
Criticism of others	7.12 (2.00)	[6.68, 7.57]	6.80 (2.07)	[6.46, 7.15]	1.13	0.262	0.16
Acting-out hostility	4.80 (2.63)	[4.21, 5.39]	4.12 (2.21)	[3.74, 4.49]	1.96	0.052	0.28
Paranoid hostility	2.59 (2.05)	[2.13, 3.04]	2.33 (2.17)	[1.96, 2.69]	0.89	0.375	0.12
Self-criticism	3.74 (2.09)	[3.27, 4.20]	3.85 (2.06)	[3.50, 4.19]	−0.38	0.706	−0.05
Guilt	2.44 (1.58)	[2.09, 2.79]	2.31 (1.64)	[2.04, 2.59]	0.56	0.577	0.08
Total Hostility	20.69 (8.11)	[18.88, 22.49]	19.41 (7.47)	[18.15, 20.66]	1.16	0.249	0.16

**Table 3:** Comparison of hostility scores with normative data.

Subscale	ICU Relatives (M ± SD)	Normative Sample (M ± SD)
Criticism of others	6.8–7.1 ± 2.0	5.3–5.7 ± 1.8
Acting-out hostility	4.1–4.8 ± 2.4	3.2–3.5 ± 1.9
Paranoid hostility	2.0–2.7 ± 1.7	2.1–2.3 ± 1.6
Self-criticism	Elevated	Normal range
Guilt	Elevated	1.9 ± 1.3
Total hostility	High	16–17 ± 4.5

Note. Normative values are drawn from population-based reference data reported by Caine et al. (1967) and Dragioti et al. (2012) [16,19]. Since neither source reported significant sex differences in hostility subscales, male and female normative scores were averaged for comparability with the present sample.

**Table 4:** Multivariate Regression Analysis of Hostility Subscales.

Subscale	Predictor	Beta	95% CI	*p*
criticism of others	Age	−0.009	[−0.033, 0.014]	0.426
criticism of others	Gender	0.332	[−0.235, 0.900]	0.250
criticism of others	Education	−0.446	[−1.000, 0.109]	0.114
acting-out hostility	Age	−0.029	[−0.055, −0.002]	0.035
acting-out hostility	Gender	0.735	[0.083, 1.387]	0.027
acting-out hostility	Education	−0.686	[−1.323, −0.049]	0.035
paranoid hostility	Age	0.004	[−0.019, 0.027]	0.752
paranoid hostility	Gender	0.226	[−0.342, 0.793]	0.435
paranoid hostility	Education	−1.227	[−1.782, −0.672]	<0.001
self-criticism	Age	−0.002	[−0.025, 0.021]	0.881
self-criticism	Gender	−0.129	[−0.690, 0.432]	0.651
self-criticism	Education	−1.022	[−1.570, −0.474]	<0.001
Guilt	Age	0.005	[−0.013, 0.023]	0.558
Guilt	Gender	0.098	[−0.343, 0.538]	0.663
Guilt	Education	−0.719	[−1.149, −0.289]	0.001
Total Hostility	Age	−0.031	[−0.116, 0.054]	0.477
Total Hostility	Gender	1.261	[−0.818, 3.340]	0.233
Total Hostility	Education	−4.100	[−6.132, −2.069]	<0.001

Note. Gender coded as male = 1, female = 0; Education coded as higher = 1, primary/secondary = 0; Age entered as continuous (per year). *p*-values < 0.001 shown as ‘<0.001’.

## Data Availability

The original contributions presented in this study are included in the article. Further inquiries can be directed to the corresponding authors. The data are not publicly available due to due to privacy and ethical restrictions.
